# Epidemiology of Shiga toxin producing *Escherichia coli *in Australia, 2000-2010

**DOI:** 10.1186/1471-2458-12-63

**Published:** 2012-01-21

**Authors:** Hassan Vally, Gillian Hall, Amalie Dyda, Jane Raupach, Katrina Knope, Barry Combs, Patricia Desmarchelier

**Affiliations:** 1 National Centre for Epidemiology and Population Health, ANU College of Medicine, Biology and Environment, The Australian National University, Canberra, Australia; 2 School of Public Health and Human Biosciences, La Trobe University, Melbourne, Australia; 3 Medical School, ANU College of Medicine, Biology and Environment, The Australian National University, Canberra, Australia; 4 South Australian Department of Health, Government of South Australia, Canberra, Australia; 5 OzFoodNet, Australian Department of Health and Ageing, Canberra, Australia; 6 OzFoodNet, Western Australian Department of Health, Canberra, Australia; 7 Food Safety Consultant, Food Safety Principles, Brisbane, Australia

## Abstract

**Background:**

Shiga toxin-producing *Escherichia coli *(STEC) are an important cause of gastroenteritis in Australia and worldwide and can also result in serious sequelae such as haemolytic uraemic syndrome (HUS). In this paper we describe the epidemiology of STEC in Australia using the latest available data.

**Methods:**

National and state notifications data, as well as data on serotypes, hospitalizations, mortality and outbreaks were examined.

**Results:**

For the 11 year period 2000 to 2010, the overall annual Australian rate of all notified STEC illness was 0.4 cases per 100,000 per year. In total, there were 822 STEC infections notified in Australia over this period, with a low of 1 notification in the Australian Capital Territory (corresponding to a rate of 0.03 cases per 100,000/year) and a high of 413 notifications in South Australia (corresponding to a rate of 2.4 cases per 100,000/year), the state with the most comprehensive surveillance for STEC infection in the country. Nationally, 71.2% (504/708) of STEC infections underwent serotype testing between 2001 and 2009, and of these, 58.0% (225/388) were found to be O157 strains, with O111 (13.7%) and O26 (11.1%) strains also commonly associated with STEC infections. The notification rate for STEC O157 infections Australia wide between 2001-2009 was 0.12 cases per 100,000 per year. Over the same 9 year period there were 11 outbreaks caused by STEC, with these outbreaks generally being small in size and caused by a variety of serogroups. The overall annual rate of notified HUS in Australia between 2000 and 2010 was 0.07 cases per 100,000 per year. Both STEC infections and HUS cases showed a similar seasonal distribution, with a larger proportion of reported cases occurring in the summer months of December to February.

**Conclusions:**

STEC infections in Australia have remained fairly steady over the past 11 years. Overall, the incidence and burden of disease due to STEC and HUS in Australia appears comparable or lower than similar developed countries.

## Background

Since 1982 gastroenteritis from Shiga toxin-producing *Escherichia coli *(STEC), an *E.coli *strain with the capacity to produce a cytotoxin similar to that produced by *Shigella *spp., has been identified as a significant health problem in the developed world [1,2]. Infections with STEC, of which *E.coli *O157 is the most well known serotype, have been recorded in many regions- including North America, Western Europe, Japan, Central and South America, the Middle and Far East, Africa and Australia [3,4]. Infections by STEC are characterized by abdominal cramps and acute bloody diarrhoea [5]; however, more serious sequelae may also result, including haemolytic uraemic syndrome (HUS) and associated complications, which can lead to kidney failure and death in some individuals [3,6].

The majority of illness due to STEC appears to be sporadic, although large outbreaks have been reported. Cattle and sheep are the main reservoirs of STEC and it is generally believed that the major transmission route is foodborne, with the source of infection being the contamination of food with animal faeces [7]. Foods that have been found to be associated with infection are poorly handled or inadequately cooked beef hamburgers and ground beef [8,9], inadequately preserved meats [10,11], raw or inadequately pasteurised dairy products [12,13] and juices [14,15], contaminated sprouted seeds [16,17] and fresh produce [18,19]. In addition to the foodborne transmission route, however, there have been some large waterborne outbreaks [20] and other transmission routes such as person-to-person and animal-to-person also appear to play an important role, particularly in sporadic cases [7,21]. Despite the fact that the incidence of STEC infections appears to be much lower than the incidence of other bacterial enteric infections caused by *Salmonella *spp. and *Campylobacter *spp., the illness caused can be severe and thus STEC is considered a significant challenge to public health [22]. The extent of the threat posed by infection with STEC and other Shiga toxin producing *E. coli *pathotypes has been most recently highlighted by the large outbreak of bloody diarrhoea and HUS associated with the rare *E.coli *serotype O104:H4 [23]. This geographically widespread and severe outbreak caused by contaminated sprouts resulted in over 4000 cases and 50 deaths in Germany and 15 other countries.

In this article we describe the epidemiology of STEC in Australia using the latest available national and state and territory surveillance data, as well as data on serotypes, hospitalizations, mortality and outbreaks.

## Methods

### Australian notifiable disease surveillance data for STEC and HUS

Information on the incidence of STEC infections and HUS in Australia was obtained from the National Notifiable Diseases Surveillance System (NNDSS) which is a collation of data from state and territory health departments. Notification of laboratory confirmed STEC and clinically diagnosed HUS to health departments has been mandatory in Australia in all jurisdictions for the 11 year period, except Queensland and Western Australia where it became notifiable in 2001 [24].

The national notifiable diseases case definition for STEC illness requires laboratory evidence only, which involves either the isolation of STEC from faeces, the detection of Shiga toxin from a clinical isolate of *E. coli*, or the identification of the gene (*stx*_*1 *_and/or *stx*_*2*_) associated with the production of Shiga toxin in *E. coli *by nucleic acid testing on an isolate or raw bloody diarrhoea [24]. The national case definition for HUS requires the presence of acute microangiopathic anaemia on peripheral blood smear (schistocytes, burr cells or helmet cells) and the presence of at least one of: acute renal impairment (haematuria, proteinuria or elevated creatinine level) or thrombocytopaenia, particularly during the first 7 days of illness [25].

Using data obtained from the NNDSS website [26], notification rates for both STEC infections and HUS were examined for the 11 year period 2000-2010. HUS rates included both diarrhoea associated HUS and non-diarrhoea associated HUS.

### South Australian surveillance data for STEC and HUS

South Australia is the state with the most comprehensive surveillance for STEC illness in Australia. This was initiated after a serious outbreak of paediatric HUS due to contaminated mettwurst in 1995 [27]. In this State, all stools are tested for presence of STEC where there is evidence of blood in the stool, a clinical history of blood in the stool, or where the requesting doctor queries STEC infection or HUS [28]. Initially stools are screened using real time PCR for the presence of *stx*_*1 *_and *stx*_*2 *_genes only and positive specimens are tested with a multiplex PCR for virulence genes *stx*_*1*_, *stx*_*2*_, *eae*, *hlyA*, *saa, and serotype genes O111, O157, O113 *[29]. All faecal samples that screen positive for *stx*_*1 *_and *stx*_*2 *_are also cultured, and with few exceptions, STEC isolates are obtained, all of which are serotyped.

### National OzFoodNet datasets

The Commonwealth Department of Health and Ageing established the OzFoodNet network to enhance surveillance for foodborne disease across Australia in 2000 [30]. OzFoodNet network partners include the National Centre for Epidemiology and Population Health at The Australian National University, the Public Health Laboratory Network, and all eight States and Territories of Australia. OzFoodNet maintains a number of databases with comprehensive information on foodborne disease in Australia.

#### National OzFoodNet enhanced data on serotypes

National data on serotype distribution was analysed using OzFoodNet enhanced data on serotypes for 2001 to 2009. It is important to note that there is some variation in methods of testing and reporting on serotypes across the different states and territories that may influence the serotypes reported and included in this database. OzFoodNet enhances this serotype data each year before producing their annual report by checking for completeness and accuracy. Changes made to the data by OzFoodNet for their annual reports may not always be incorporated into state and territory surveillance systems. Similarly, state and territory data may be updated at a later time following the publication of the OzFoodNet annual report and these changes will not be reflected in the OzFoodNet data. Small differences in numbers may hence result between OzFoodNet data and the datasets maintained by the individual states and territories.

#### National OzFoodNet outbreak register data

The OzFoodNet Outbreak Register is a collation of information on all outbreaks of gastroenteritis since 2001. Data describing all STEC outbreaks that had occurred in Australia between 2001 and 2009 were examined. Outbreak register data was cross checked with annual reports and adjusted accordingly.

### Hospital and mortality data for STEC and HUS

The National Hospital Morbidity Database [31] was examined for episodes of admission for STEC illness (ICD 10 codes A04.0 toA04.4) and HUS (ICD-10 code D59.3) where these illnesses were coded as the principal diagnosis (main reason for admission) during the 9 year period 1999/2000 to 2007/08. The rubrics for ICD-10 AM [32] codes used to identify hospitalisations and deaths for probable STEC infection and HUS were the following: A04.0: Enteropathogenic *E.coli *infection; A04.1 Enterotoxigenic *E.coli *infection; A04.2: Enteroinvasive *E.coli *infection; A04.3: Enterohaemorrhagic *E.coli *infection; A04.4: other intestinal *E.coli *infections; and D59.3: Haemolytic uraemic syndrome. While A04.3 is the most appropriate description for STEC infection, it was likely that other related codes were also used during coding of STEC infection admissions and therefore the results tables refer to episodes coded to the wider range of codes. The hospitalization data are recorded as episodes of admission and do not give information on repeat admissions for the same person. The data are provided as financial years (July to June the following calendar year) and so are not strictly comparable with data from other datasets provided by calendar year.

Mortality unit record data were obtained from the Australian Bureau of Statistics and examined for the period 2000-2007. The number of deaths was identified where HUS (ICD-10 code D59.3) was categorized as the main cause of death, as a contributing cause of death, or as a significant condition related to death. Deaths where there was no HUS but there was likely STEC infection (ICD-10 codes A04.0 to A04.4) were also identified.

### Population data

Annual rates were calculated using numerator data of the number of annual notifications, hospitalizations or deaths and denominator data of the appropriate midyear population from the Australian Bureau of Statistics [33,34].

### Ethics

Ethics approval for the analysis of unit record data was obtained from the Australian National University Human Research Ethics Committee.

## Results

### Incidence of STEC illness

For the 11 year period 2000 to 2010, the overall annual Australian notification rate of STEC illness was 0.4 cases per 100,000 per year, with annual rates ranging from 0.2 to 0.6 per 100,000 per year (Table 1). Overall, the number and rates of notifications of STEC nationally appeared to increase slightly between 2000 and 2010, with the annual rate being approximately 0.2 cases per 100,000 per year between 2000 and 2004, and approximately 0.5 cases per 100,000 per year between 2005 and 2010. In South Australia, the jurisdiction with the most comprehensive surveillance in the country, the notification rate was considerably higher over this period, with 2.4 cases per 100,000 per year (ranging from 1.8 to 3.8 cases per 100,000 per year). The notification rate in South Australia appeared to be fairly steady over this period, notwithstanding a spike in 2009 due to a number of outbreaks which occurred in this state during this year.

**Table 1:** Notification of human STEC illnesses in Australia and in the state of South Australia, 2000 to 2010

Year	N, national notifications	Rate, National notifications per 100,000/year	N, South Australia notifications	Rate, South Australia notification per 100,000/year
2000	37	0.2	33	2.2
2001	46	0.2	27	1.8
2002	59	0.3	39	2.5
2003	52	0.3	37	2.4
2004	51	0.2	30	1.9
2005	84	0.4	39	2.5
2006	70	0.3	35	2.2
2007	106	0.5	40	2.5
2008	107	0.5	39	2.4
2009	130	0.6	62	3.8
2010	80	0.4	32	1.9

**Total**	**822**	**0.4**	**413**	**2.4**

Source: Data extracted from National Notifiable Diseases Surveillance System website [26]

The examination of the annual number of notifications across each of the jurisdictions indicated that the Australian Capital Territory and Tasmania had the lowest numbers of cases and lowest rates with only 1 and 2 notifications over the eleven year period between 2000 and 2010, respectively (Table 2). In contrast, the jurisdiction with the highest number of notifications by a fair margin was South Australia 413 notifications. The number of notifications remained steady in most of the jurisdictions, however, in New South Wales, Victoria and Queensland, there appeared to be a slight increase in the number of notifications from around the middle part of the period.

**Table 2:** Number of notifications of STEC illness by year and jurisdiction, 2000 to 2010

YEAR	NSW	VIC	ACT	WA	QLD	SA	TAS	NT
2000	1	3	0	NN*	NN*	33	0	0
2001	1	1	0	3	14	27	0	0
2002	6	5	0	4	5	39	0	0
2003	3	3	0	3	6	37	0	0
2004	5	4	0	0	10	30	0	0
2005	16	8	0	12	9	39	2	0
2006	10	5	0	3	15	35	0	2
2007	23	13	1	2	24	40	0	3
2008	19	11	0	0	38	39	0	0
2009	21	16	0	6	24	62	0	1
2010	10	12	0	8	18	32	0	0

**TOTAL**	**115**	**81**	**1**	**41**	**163**	**413**	**2**	**6**

**Yearly Rate/100,000**	**0.17**	**0.14**	**0.03**	**0.18**	**0.37**	**2.4**	**0.04**	**0.07**

*NN= Not notifiable

Source: Data extracted from National Notifiable Diseases Surveillance System website [26]

### Age and sex distribution of STEC

Sixteen percent of all STEC cases nationally occurred in children < 5 years and the national notification rate was highest for this age group at 0.9 cases per 100,000 per year with all other age groups ranging from 0.3 to 0.5 cases per 100,000 per year (Table 3). In the adult age range 15-59 years there were slightly more notifications for females compared to males. Age/sex distributions across all of the states in Australia were fairly similar (data not shown).

**Table 3:** Notifications STEC in Australia 2000 to 2010, by age and sex

	Males	Females	Total	Rate, notifications per 100,000/year
< 5 years	63	62	125	0.9
5 - 14 years	71	55	126	0.4
15 - 59 years	157	205	362	0.3
60+ years	80	128	208	0.5

**Total**	**371**	**450**	**822**	**0.4**

Source: Data extracted from National Notifiable Diseases Surveillance System website [26]

### Incidence and distribution of STEC serogroups

**Figure 1:**
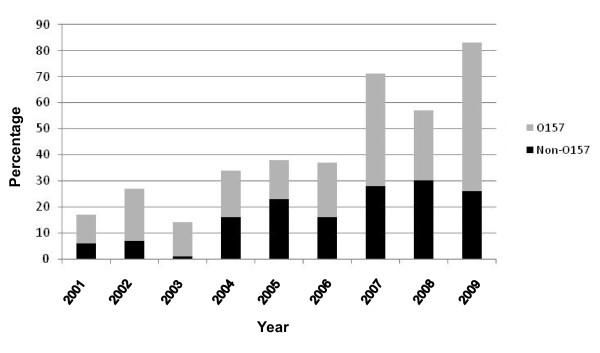
**Proportion STEC O157 and non O157 isolates (of those with a known serotype) reported in Australia between 2001 and 2009**.

Between 2001 and 2009, 504 of 708 (71.2%) STEC illness notifications in Australia had serotyping information and results were recorded in the OzFoodNet database containing enhanced data on serotypes. Although 116 isolates were unable to be typed for various reasons, for the remaining 388 a known serotype was established, and of these 225 (58%) were found to be O157 strains, with 163 (42%) non O157 STEC strains. Among those with an identified serotype, the proportion of strains with O157 serotype over this period ranged from a low of 39% (in 2005) to a high of 74% (in 2002), with no obvious trend apparent (Figure 1). The overall average annual rate of notifications of confirmed STEC O157 illnesses in Australia between 2001 and 2009 was 0.12 cases per 100,000 per year.

In South Australia, where there is more extensive STEC testing using sensitive genetic testing methods compared with other states, 460 cases of STEC infection were notified between 1997 and 2009 and 281 (61%) had serotype information [28]. Among the cases with serotype information, O157 strains accounted for 140 cases (50%). The annual rate of notifications of STEC O157 for South Australia was 0.7 cases per 100,000 per year between 1997 and 2009. Based on 2001-2009 OzFoodNet data amongst non O157 STEC infections with serotype information, O111 (13.7% of serotyped strains) and O26 (11.1%) were found to be the most represented serogroups. Serotypes O113, O55 and O86 also contributed to non O157 notifications. The full spectrum of serogroups detected in Australia between 2000 and 2009 is summarized in Table 4.

**Table 4:** Serotypes detected in Australia from OzFoodNet enhanced data 2001-2009 (n = 504 isolates)

Percentage of isolates with a known serotype	Serotypes
Common* (> 1%)	O157 (58%), O111 (13.7%), O26 (11.1%), O113 (3.6%), O55 (1.3%), O86 (1.0%)

Uncommon* (< 1%)	O2, O5, O28, O49, O77, O88, O103, O112, O124, O128, O130, O153, O145, O166, O172, O174, O178, O141, OR, O123, O165, ONT:H2/H7/H18/H19/H49

Source: OzFoodNet Unpublished Data, 2010

### Incidence of haemolytic uraemic syndrome in Australia

There were a total of 169 notifications of HUS notified nationally to NNDSS in the period 2000 to 2010 as shown by year and jurisdiction in Table 5, and by age and sex in Table 6. The overall average annual rate of notification for HUS in the 11 year period 2000-2010 was 0.07 cases per 100,000 per year. The notification rates for HUS ranged from below 0.05 cases per 100,000 per year in the Australian Capital Territory, Tasmania and Western Australia to a high of 0.13 cases per 100,000 per year in New South Wales. The number of notifications increased in New South Wales from 2003 to 2008 but other states remained stable. Nationally, there were nearly twice as many females (n = 29) than males (n = 15) notified with HUS in the age group 16-60 year, and children under 5 years had the highest rate of HUS at 0.49 cases per 100,000 per year (Table 6). Data were not available for the number of HUS cases that were diarrhoea associated or STEC positive.

**Table 5:** Number of notifications of HUS* by year and jurisdiction 2000 to 2010

YEAR	NSW	VIC	ACT	WA	QLD	SA	TAS	NT	Total
2000	9	3	0	1	3	1	0	0	17

2001	2	0	0	0	0	1	0	0	3

2002	7	4	0	0	1	0	0	1	13

2003	5	4	0	1	1	3	0	1	15

2004	9	1	0	1	1	3	0	1	16

2005	11	3	0	1	2	1	2	0	20

2006	11	1	0	0	1	1	0	0	14

2007	13	3	1	0	1	1	0	0	19

2008	17	4	0	0	7	2	0	1	31

2009	4	2	0	0	1	4	0	1	12

2010	1	3	0	0	2	0	0	0	8

Total	**91**	**28**	**1**	**4**	**22**	**17**	**2**	**4**	**169**

Yearly Rate/**100,000**	**0.13**	**0.05**	**0.03**	**0.02**	**0.05**	**0.10**	**0.04**	**0.05**	**0.07**

*diarrhoea and non diarrhoea associated Source: Data extracted from National Notifiable Diseases Surveillance System website [26]

**Table 6:** Notifications of HUS* in Australia 2000 to 2010 by age and sex

Age Group	Males	Females	Total	Rate notifications per 100,000 per year
< 5 years	33	38	71	0.49
5 - 15 years	16	15	31	0.10
16 - 60 years	15	29	44	0.03
60+ years	9	14	23	0.06

**Total**	**73**	**96**	**169**	**0.07**

*diarrhoea and non diarrhoea associated

### Hospitalisations and deaths due to STEC infection

National hospitalisation data indicated that there were 152 admissions for STEC infection as a principal diagnosis over the 9 year period between 1999/2000 and 2007/08, giving a rate of 0.08 admissions per 100,000 per year. There were three deaths from STEC infection where HUS was not recorded as a co-condition in the period 2000-2007, with all of these occurring in people over 60 years of age. However, South Australian notifiable surveillance data from a 13 year period 1997-2009 indicated that 33% (150/460) of STEC cases were admitted to hospital for any complication, including HUS, giving a much higher population rate of approximately 0.8 cases per 100,000 per year [28]. The rate was greater in the last 5 years 2005-2009 at about 1 per 100,000 per year.

### Hospitalisations and deaths due to HUS

There were 670 hospital admissions recorded in the hospitalisation database nationally for HUS between 1999/2000 and 2007/2008, indicating a rate of hospitalisation of 0.37 per 100,000 per year (Table 7), which is a considerably higher rate than the number of new cases of HUS reported to NNDSS for almost the same period (0.08 cases per 100,000 per year). Over time the annual hospitalisation rates for HUS appear to have increased slightly.

**Table 7:** Hospitalisation episodes^1 ^due to HUS* in Australia 1999/2000 to 2007/2008

Year	Number of Hospitalisations	Hospitalisations per 100,000 per year
1999/2000	47	0.25
2000/01	64	0.35
2001/02	50	0.25
2002/03	82	0.41
2003/04	45	0.22
2004/05	110	0.54
2005/06	91	0.44
2006/07	76	0.36
2007/08	105	0.49

**Total**	**670**	**0.37**

^1^Principle diagnosis (not including additional diagnoses) *diarrhoea and non diarrhoea associated HUS included in this table

National hospitalisation episode rates for HUS were highest for children < 5 years at 1.47 per 100,000 per year. The overall rates were similar for males (0.36 cases per 100,000 per year) and females (0.38 cases per 100,000 per year) although in the youngest age group < 5 years, there were twice as many males hospitalised (n = 116) as females (n = 56). Among people aged 16-60 years there were more hospitalisations for women (n = 189) than men (n = 100), reflecting the distribution of HUS notifications.

There were 14 deaths recorded in the Australian Mortality Database from 2000 to 2007 where HUS was the main underlying cause of death, representing a rate of 0.01 deaths per 100,000 per year. In another 18 deaths, HUS was listed as an additional contributing cause of death, or as a significant condition, but not as the main underlying cause. When all 32 deaths were counted, the rate increased to 0.02 deaths per 100,000 per year. There was no difference by sex with 16 deaths for each of males and females, and there was no apparent trend over time. Among the deaths where the main underlying cause was HUS, two were in the age group 0-4 years, three were 20-50 years, and nine were 50 = years. Among those with HUS as a contributing cause but not an underlying cause, one was 0-4 years, one was 5-9 years, five were 20-50 years, and eleven were 50 = years. The age specific death rates indicated that the under 5 year age group was particularly vulnerable (0.03 cases per 100,000 per year when all deaths are counted) reflecting the higher incidence of both STEC and HUS in this age group. The over 50 year age group was also vulnerable with a higher rate of deaths from HUS (0.04 cases per 100,000 per year).

From national notifiable data there were 117 notifications of HUS in the period 2000-2007 and from the national mortality data 14 deaths where HUS was the main underlying cause of death, suggesting that 12.0% of HUS cases died. However, as notification data are not linked individually to mortality data it is likely that some deaths from HUS are for cases notified in years outside the given period if HUS led to chronic problems lasting a number of years.

Among the 460 STEC cases reported to South Australia's notifiable diseases surveillance data in the 13 year period 1997-2009, there were 14 reported cases of HUS, giving an estimate of 3% of STEC cases developing HUS. The annual incidence of HUS associated with STEC infection in South Australia was 0.1 cases per 100,000 per year. There was no particular serovar strongly associated with these cases. There were no deaths from HUS reported in South Australia over this period [28].

### Seasonality of STEC and HUS

**Figure 2:**
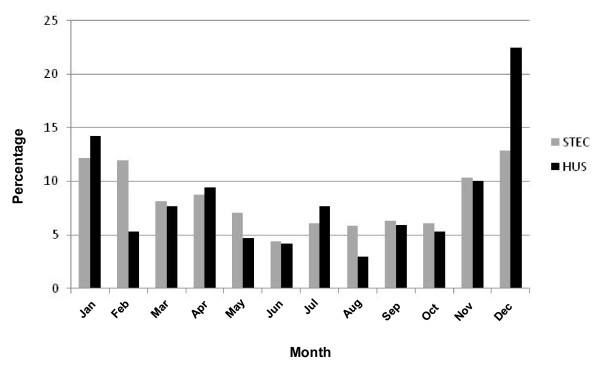
**Seasonal distribution of STEC and HUS notifications in Australia: percentage of cases by month of onset, 2000-2010**.

The distribution of STEC and HUS cases nationally were seasonal with a larger proportion of reported cases occurring in the summer months of December to February and lower numbers in winter (Figure 2).

### Burden, incidence and characteristics of STEC outbreaks

Between 2001 and December 2009 there were 11 outbreaks which were due to STEC, which collectively affected 117 people (Table 8). Most outbreaks were small, with a median size of six people affected. There were 29 people (25%) hospitalized and no deaths due to STEC infections from outbreaks over this period.

**Table 8:** Outbreaks of STEC reported to the OzFoodNet outbreak register 2001-2009, Australia

Year	State	Transmission mode	Number ill	Hospitalised	Setting	Food/water vehicle	STEC serotype
2002	SA	Animal-to-person	6	0	Petting zoo	Not applicable	O26:H-

2003	SA	Person-to-person	13	2	Aged Care	Not applicable	O111:H-

2004	QLD	Unknown	3	2	Unknown	Not applicable	O86:H27

2005	SA	Unknown	4	3	Community	Unknown	O111

2007	QLD	Unknown	3	1	Unknown	Not applicable	O55

2007	SA	Unknown	12	7	Community	Not applicable	O157

2008	QLD	Waterborne (including recreational water)	2	1	Camp	Tank water	Multiple serotypes

2009	multi-state	Suspected foodborne	14	5	Community	Unknown	O157:H-

2009	QLD	Person-to-person	4	0	Child care	Not applicable	OR:H7

2009	SA	Foodborne	31	5	Camp	Potato salad	O157

2009	multi-state	Unknown	25	3	Community	Not applicable	O157:H7

Theses outbreaks were reported from South Australia (n = 5), Queensland (n = 4) and two involved a number of states; with outbreaks occurring in a variety of settings. Only two outbreaks were due to foodborne or suspected foodborne transmission, and for only one of these could a particular food vehicle be identified (potato salad with homegrown parsley). Two outbreaks were suspected to be due to person-to-person transmission, one due to animal-to-person transmission and one was thought to be due to waterborne transmission from tank water (as published by McCall et al. [35]). The mode of transmission in the remaining five outbreaks remained unknown. One of these is the outbreak described by Hanna et al. [36] as being due to possible animal-to-person spread. A number of different serotypes were isolated from these outbreaks, with *E.coli *O157 thought to have been the infecting serotype in four (36.4%) outbreaks. For one outbreak multiple serotypes were involved.

## Discussion

The average annual notified rate of STEC illness over the past 11 years (2000-2010) in Australia using NNDSS data was estimated to be 0.4 cases per 100,000 per year, with an average rate of confirmed O157 STEC illness of 0.12 cases per 100,000 per year. Although it is difficult to see a clear trend from these national data, rates look to have increased slightly since 2000. Whilst this may represent a real increase in the STEC illness rate, there is also evidence that the increase may be linked to an increase in the number of stool samples tested and changes in laboratory methods used to detect STEC (unpublished observations).

Variation in rates across states may also be partly related to variation in testing practices. The number of stools tested for STEC varies considerably between different states of Australia and this appears to be correlated with STEC notifications rates. The laboratory tests used to detect STEC also varies between jurisdictions, with some using sorbitol MacConkey agar to culture STEC and others incorporating PCR for detection of *stx1 *and *stx2 *genes, which is considered the more sensitive method [37]. The Australian state that tests the most number of stools is South Australia even though it does not have a large population compared with many of the other states of Australia. In this jurisdiction, a single reference laboratory tests specimens that include those with evidence of blood in the stool, where there is a clinical history of blood in the stool, or where the requesting doctor queries STEC infection or HUS. The specimens tested are referred from other private and public health laboratories and so this jurisdiction is considered to provide Australia with amongst the most robust estimates internationally for STEC incidence in bloody diarrhoea [24,28]. However, despite the quality of the surveillance in this state it is not entirely clear if STEC rates and epidemiology can be extrapolated from South Australia to other regions of the country, as Australia is also a very large country with differing geography, climate and demographics.

South Australian surveillance data showed that the 11 year average rate for STEC infections was 2.4 cases per 100,000 per year, which is over 6-fold greater than the national rate over this period. South Australian STEC incidence rates have remained fairly steady over a considerable period, apart from the increase in 2009 due to several outbreaks in this jurisdiction in that year. As there have been no major changes in surveillance practices in South Australia over the last 15 years, this supports the notion that there has been no real change in the rate of STEC infections in Australia over this period.

Surveillance practices also vary considerably between countries and therefore caution is required when comparing STEC incidence rates between countries. In particular, laboratory testing practices vary considerably, especially the practices for screening stools for blood and the extent to which genetic testing methods are utilised, and in addition, the extent to which testing for all serotypes occurs. In Australia, testing for non O157 serotypes is an important component of surveillance with most jurisdictions conducting STEC testing also using methods to detect non O157 serotypes [24]. However, testing for non O157 serotypes does not occur in many countries, with most focussing mainly on the detection of O157 strains [24,38-41]. In addition, case definitions are not the same across countries and some reports are for 'confirmed' cases only, while others include 'probable' cases. Some countries have passive or voluntary reporting, while others are mandated by law or actively pursued. Furthermore, multiple STEC infections in one person may be reported as separate infections or as one case. Even within countries there is variation in testing and reporting. For example, in the Netherlands only some parts of the country test for non-O157 [38]. Also, in the United States, only 7% of 428 clinical laboratories surveyed used enzyme immunoassay, a non-culture method for the detection of non O157 cases [40].

Bearing in mind the need for caution regarding interpretation, Australian rates appear to be lower than countries in Europe that have similar surveillance practices and report confirmed cases of both O157 and non O157 STEC. Even the higher rates obtained from the South Australian surveillance system are comparable to rates reported in these countries. The Community Summary Report on the European Union in 2008 [38] gives the incidence of all STEC infections in 2008 in Austria, Belgium and Norway as 1 case per 100,000 or less; and Denmark, Sweden and Ireland as 2.9, 3.3 and 4.8 cases per 100,000, respectively. New Zealand, Australia's nearest neighbour, reported STEC in 2009 at a rate of 3.3 cases per 100,000 per year [42]. In many countries the vast majority of reported STEC cases were serotype O157, including Scotland, Ireland, the United Kingdom as a whole, the United States and Canada. In Scotland in the period 1998-2007, the annual average incidence of O157 infections was 4.3 cases per 100,000 per year [39]. In Ireland in 2009, O157 incidence was 3.9 cases per 100,000 per year [43] whilst in the whole of the United Kingdom in 2008 it was reported to be 1.9 cases per 100,000 per year [38]. In the United States, between 2000- 2006, data gathered from eight FoodNet surveillance sites gave an incidence of O157 of 1.5 cases per 100,000 per year [44]. In Canada in 2007 the incidence of O157 was 2.9 cases per 100,000 per year [41]. It should be noted that all of these rates are higher than the incidence rate for confirmed O157 for South Australian of 0.7 cases per 100,000 per year in the period 1997-2009.

The overall incidence of HUS in Australia in the 11 year period 2000-2010 was 0.07 per 100,000 per year with children under 5 years having the highest rate of 0.49 cases per 100,000 per year. It is important to recognise that HUS can be due to various causes and not all cases are secondary to STEC infection. An estimate by a group of Australian foodborne disease experts in 2005 suggested that about 50% of cases were secondary to STEC infection [45]. Other evidence suggests the proportion could be as high as 88%, with 139 out of 160 HUS cases reported to the Australian Paediatric Surveillance Unit between 1994 and 2001 being diarrhoea associated [46].

National hospital separation data showed more episodes of hospitalisation for HUS than the number of cases notified to national surveillance. Firstly, it is likely that patients with HUS may have multiple hospital admissions, especially as they may be transferred to specialist units and so are recorded as more than one hospital admission episode. Furthermore, any cases proceeding to dialysis may require many admissions. Cases from earlier years that developed chronic renal failure due to HUS may still appear in this hospitalisation data in this time period for treatment. It is also possible that some hospitalised cases are not reported to the national surveillance system. There may be some cases that were not STEC but other types of *E.coli *infection included in the codes used to identify episodes of hospitalisations attributed to STEC. On the other hand, there are also likely to be other STEC infections coded to diarrhoea of presumed infectious origin. A validation study linking notifications, hospitalisations and deaths would be valuable to clarify this issue.

Worldwide, case control studies of varying sizes and rigour have been conducted aiming to examine risk factors for sporadic O157 STEC infection. In many of the larger well conducted North American studies a significant association between illness and the consumption of hamburgers, pink or undercooked hamburgers, pinkish ground beef, undercooked meat, or barbequed food, has been demonstrated [47-51]. In addition to this, eating in restaurants or fast food restaurants were also identified as risk factors [48,49]. Living or working or visiting a cattle farm [47] and visiting a farm with cows [48] were also both found to be strongly associated with O157 STEC infection in two of the larger United States studies completed. Drinking untreated surface water, drinking well water, or swimming in a pond were other environmental exposures associated with infection in these North American studies. Other case control studies have indicated the association between illness and contact with animal faeces [52], consumption of raw milk [53] and consumption of cold cooked sliced meats [54]. Evidence of household transmission has also been obtained, with an association between STEC infection and the presence in the household of a child under the age of 2 years [48], a child with diarrhoea [55], or anyone with diarrhoea [49].

In Australia, two case control studies have examined risk factors for sporadic STEC infection. In South Australia in 2002 cases were more likely than controls to have eaten berries, including strawberries, blueberries and blackberries, in the 10 days preceding illness [56]. This finding is interesting, although the study was small and this finding needs to be interpreted with caution. Another Australian study [57] recruited 114 cases and risk factors for infection were analysed for those infected with O157 serotypes and non-O157 serotypes of STEC. Risk factors for O157 infection in this study included eating hamburgers, visiting restaurants, having previously used antibiotics, having direct contact with red meat through occupational exposures or having another member of the household having occupational exposure to red meat. The risk factors for O157 infection in this Australian study were largely similar to those reported in international studies; however, consumption of hamburgers and ground beef has not been previously thought to be an important cause of STEC infections in Australia so this finding is of considerable interest. Risk factors for non O157 infection included having eaten sliced chicken meat or corned beef from a delicatessen, having camped in the bush, having eaten catered meals, or having had family occupational exposure to animals. The grouping of all non O157 serotypes in this study into one group assumes that different serovars have similar characteristics and risk factors which is a limitation; however, the identification of different risk factors for O157 and non O157 STEC almost certainly reflects differing reservoirs for these serogroups in Australia.

STEC outbreaks appear to affect less people in Australia compared to many countries. A total of 117 cases were reported as part of 11 outbreaks in Australia as a whole in the nine year period between 2001-2009, a crude rate of 0.5 outbreaks per million population per year and representing approximately 15% of all STEC cases reported. This is much lower than the proportion of outbreak cases in some other comparable countries. For example, in Ireland, 50% of cases in 2009 were related to 42 outbreaks in that year [43]. In New Zealand between 2006 and 2009 there were 19 outbreaks of STEC, three of them foodborne [42,58]. In 2008 in the European Union, there were 75 foodborne outbreaks of STEC and other pathogenic *E.coli*, up from 65 outbreaks in 2007 [59]. The largest numbers of outbreaks reported from a single country were in Germany (28 outbreaks) and Austria (11 outbreaks) with only four reported from the United Kingdom. In the United States in 2007 there were 40 outbreaks of STEC [60]. In the United States, ground beef appears has posed the most significant public health risk and has been the food most commonly implicated in reported STEC outbreaks, although over time there have been major outbreaks attributed to other sources, such raw milk products, fresh produce, juice, sprouted seeds and spinach [12-19,61]. In 2007, five out of six multi-state outbreaks of *E.coli *O157:H7 were due to ground beef [60]. Many of the outbreaks found to be due to ground beef over the years have been extremely large in size and have affected up to many hundreds of individuals [9,62]. The majority of these outbreaks were most likely to have been due to beef where contamination was disseminated in product during the grinding process and then undercooked during preparation [3]. It is important to mention that in addition to the 11 outbreaks in Australia reported between 2001 and 2009, there was also a significant STEC outbreak in South Australia in 1995, with 23 HUS cases in children and one death associated with the consumption of mettwurst, a fermented but uncooked meat product [27].

The epidemiology of STEC serotypes suggests O157 strains have been less dominant in Australia than the United States, England, Wales, Scotland and Japan, both in terms of sporadic cases, and particularly as a cause of outbreaks. As mentioned earlier, in many countries, including the United States [40], O157 has been the focus of testing regimes, and this goes some way towards explaining the predominance of this strain in surveillance data from these countries. In Australia, testing for non O157 serotypes does occur routinely and has shown that O111, O26, O113, O55 and O86 strains are also commonly associated with STEC illnesses.

The burden of illness associated with STEC and HUS is considerable. A study exploring the economic costs of STEC infection in South Australia between 2003 and 2006 found that of the 3-7% of sporadic STEC patients who developed HUS, 40% had ongoing medical issues [63]. In this study, 19 out of 43 STEC cases were admitted to hospital (44%). The estimated average cost of STEC infection was AUD 3,132 per case. It is also likely that in every country, the reported burden is an underestimate and that there are cases in the community that are not tested and reported [64]. In South Australia, it has been estimated that there are around eight (95% credible interval 3-75) cases in the community for every case detected by surveillance [65]. After accounting for under reporting of STEC to surveillance, this equated to a cost of AUD 2,633,181 for Australia each year.

## Conclusions

In Australia STEC is a public health problem that needs to be addressed, as it does throughout the developed world. Whilst international comparisons of STEC infection rates are extremely difficult due to differing surveillance practices, and there is a need for caution in the interpretation of this data, the estimated incidence rates for STEC in Australia appear to be comparable or lower than the levels in similar countries. This is generally maintained even when using South Australian STEC surveillance data as a proxy for Australian STEC infection rates, with this jurisdiction having the most comprehensive surveillance in the country that is likely to be providing estimates closer to the true rate of infection for this pathogen than estimates from many other countries. In contrast to the United States and many other countries in which STEC outbreaks are a considerable problem and are primarily due to the foodborne transmission route, STEC outbreaks in Australia are less common and have been found to be less often associated with foodborne transmission. In conclusion, as surveillance and laboratory testing practices continually improve across Australia in the next few years, so will our understanding of the epidemiology of this pathogen in this country and our ability to target interventions.

## Abbreviations

ACT: Australian capital territory; HUS: Haemolytic uraemic syndrome; ICD: International classification of diseases; NNDSS: National notifiable diseases surveillance system; NSW: New South Wales; NT: Northern territory; SA: South Australia; STEC: Shiga toxin-producing *Escherichia coli*; TAS: Tasmania; VIC: Victoria; WA: Western Australia; QLD: Queensland

## Competing interests

Funding for this project for the two senior authors HV and GH was provided by Meat and Livestock Australia.

## Authors' contributions

HV, GH and PD analysis plan, data acquisition and data analysis. AD and JR data analysis and interpretation of South Australian data. KK data analysis. All authors contributed to the writing of the manuscript and approved the final version.

## Pre-publication history

The pre-publication history for this paper can be accessed here:

http://www.biomedcentral.com/1471-2458/12/63/prepub
